# Multimodal Imaging in a Patient with Hemidystonia Responsive to GPi Deep Brain Stimulation

**DOI:** 10.1155/2017/9653520

**Published:** 2017-07-04

**Authors:** Christos Sidiropoulos, Susan M. Bowyer, Andrew Zillgitt, Peter A. LeWitt, Hassan Bagher-Ebadian, Esmaeil Davoodi-Bojd, Jason M. Schwalb, Richard Rammo, Ellen Air, Hamid Soltanian-Zadeh

**Affiliations:** ^1^Parkinson's Disease and Movement Disorders Program, Henry Ford Hospital, 6777 West Maple Road, West Bloomfield, MI, USA; ^2^Department of Neurology and Ophthalmology, Michigan State University, 804 Service Road, A 217, East Lansing, MI 48824, USA; ^3^Department of Neurology, Henry Ford Hospital, 2799 West Grand Blvd., Detroit, MI 48202, USA; ^4^Image Analysis Laboratory, Departments of Neurology and Research Administration, Henry Ford Health System, Detroit, MI 48202, USA; ^5^Department of Neurosurgery, Henry Ford Hospital, 2799 West Grand Blvd., Detroit, MI 48202, USA; ^6^Image Analysis Laboratory, Departments of Radiology and Research Administration, Henry Ford Health System, Detroit, MI 48202, USA

## Abstract

**Background:**

Dystonia is a syndrome with varied phenomenology but our understanding of its mechanisms is deficient. With neuroimaging techniques, such as fiber tractography (FT) and magnetoencephalography (MEG), pathway connectivity can be studied to that end. We present a hemidystonia patient treated with deep brain stimulation (DBS).

**Methods:**

After 10 years of left axial hemidystonia, a 45-year-old male underwent unilateral right globus pallidus internus (GPi) DBS. Whole brain MEG before and after anticholinergic medication was performed prior to surgery. 26-direction diffusion tensor imaging (DTI) was obtained in a 3 T MRI machine along with FT. The patient was assessed before and one year after surgery by using the Burke-Fahn-Marsden Dystonia Rating Scale (BFMDRS).

**Results:**

In the eyes-closed MEG study there was an increase in brain coherence in the gamma band after medication in the middle and inferior frontal region. FT demonstrated over 50% more intense ipsilateral connectivity in the right hemisphere compared to the left. After DBS, BFMDRS motor and disability scores both dropped by 71%.

**Conclusion:**

Multimodal neuroimaging techniques can offer insights into the pathophysiology of dystonia and can direct choices for developing therapeutics. Unilateral pallidal DBS can provide significant symptom control in axial hemidystonia poorly responsive to medication.

## 1. Introduction

Since dystonia syndromes arise from multiple etiologies, study of hemi-involvement potentially can offer insights into its pathophysiology. With neuroimaging techniques, such as diffusion tractography (DT) to assess structure and magnetoencephalography (MEG) to describe brain function, pathway connectivity can be studied for a better understanding of dystonia mechanisms. The former utilizes multidirectional gradients of magnetic fields to assess diffusion of water molecules and eventually help define tracts of white matter, whereas the latter is based on detection of weak brain magnetic fields and offers excellent temporal and very good spatial resolution. We present a DBS-treated hemidystonia patient whose studies provide a multiregional view of dystonia's impact.

## 2. Materials and Methods

After 10 years of persistent, slowly progressive, isolated, left axial phasic hemidystonia, involving the trunk but not the limbs, a 45-year-old right handed male presented to our center for consideration of unilateral right globus pallidus internus (GPi) deep brain stimulation (DBS). Previous trials of anticholinergics and benzodiazepines at higher doses were poorly tolerated due to side effects. MRI of the brain was normal, genetic testing was not available due to financial reasons, and family history was negative. Before surgery, MEG Coherence Source Image (CSI) analysis [1] before and after anticholinergic medication (trihexyphenidyl 2 mg) was performed during rest with eyes open and closed, using a 148 channel whole head magnetometer system in a magnetically shielded room. MRI was used to construct the cortical source model (4,000 locations distributed to represent the same amount of gray matter). MEG-CSI analysis was used to localize highly coherent activity in the brain. For each active source, the average coherence across frequencies and sources was calculated. The 10 min rest state MEG recording was divided into 80 data segments of 7.5-second duration. For each segment, neuronal source signals were identified using an independent component analysis (ICA) spatiotemporal decomposition technique developed to extract signals from distinct compact sources that demonstrate burst behavior and minimal temporal overlap with other active sources. The MR-FOCUSS source imaging technique (Moran et al., 2005) was applied to further process the source signals. Apart from the imaging algorithm, the cross-spectrum between ICA signals was calculated and a sequence of Fast Fourier Transform (FFT) spectra was applied using 0.5 s windows and 25% overlap with FFT amplitudes for 24 frequency bins of 2 Hz width between 3 and 50 Hz. The coherence between all pairings of active cortical locations within each of the 24 frequency bins was subsequently calculated along with the average coherence across frequencies and sources for each active source. Based on these calculations brain maps demonstrating areas with strong connectivity were produced.

Moreover, in the context of preoperative workup, T1-weighted and diffusion tensor imaging (DTI) data were obtained in a 3 Tesla MRI machine (GE Medical Systems, Milwaukee, USA). The T1-weighted images were acquired as a 3D volume (256 × 256 × 174) using a spoiled gradient echo sequence with TR/TI/TE = 10400/4500/300 ms, flip angle = 15°, and voxel size = 0.9375 × 0.9375 × 1 mm^3^. DTI images were acquired using echo planar imaging (EPI) with TR/TI/TE = 7500/0/76 ms, flip angle = 90°, voxel size = 0.9375 × 0.9375 × 2.6 mm^3^, 3D reconstructed volume = 256 × 256 × 67, *b*-value = 1000 s/mm^2^, 25 diffusion gradient directions, and a baseline non-diffusion-weighted image.

The pre-OP T1-weighted images were processed and labeled automatically using FreeSurfer software tool [2]. Three cortical areas (somatosensory, premotor, and motor cortex) were used in our analysis. In addition, the regions of interest (ROI) on the post-OP T1-weighted images were drawn manually by two experts (E.A., R.R.). In the right hemisphere, the ROI was the mean between the two clinically effective contacts (0 and 1 in this case) whereas the left ROI was the mirror image of the right (no DBS performed on the left side). The effective contacts were the two most ventral ones, in a double monopolar mode, with case as the anode. Then, the cortical regions and the ROIs were coregistered to the DTI space for further analysis.

The DTI images were processed using the MRtrix software tool [3]. The fiber maps were generated using probabilistic tractography starting from each dilated ROI, until 10,000 fibers (minimum length of 10 mm) were extracted. The number of fibers connecting each ROI to the three cortical areas and the entire cortex (in each hemisphere) was counted and normalized to the total number of fibers reaching the cortex. This value is defined as the DT connectivity.

## 3. Results

The patient was assessed before and one year after surgery by a movement disorders neurologist (C. S.) using the Burke-Fahn-Marsden Dystonia Rating Scale (BFMDRS). The active contacts remained at the time of postsurgery evaluation and image analysis. With DBS treatment, BFMDRS motor and disability scores both dropped by 71% (from 28/120 to 8) motor and (from 7/30 to 2) disability at one year.

**Figure 1:**
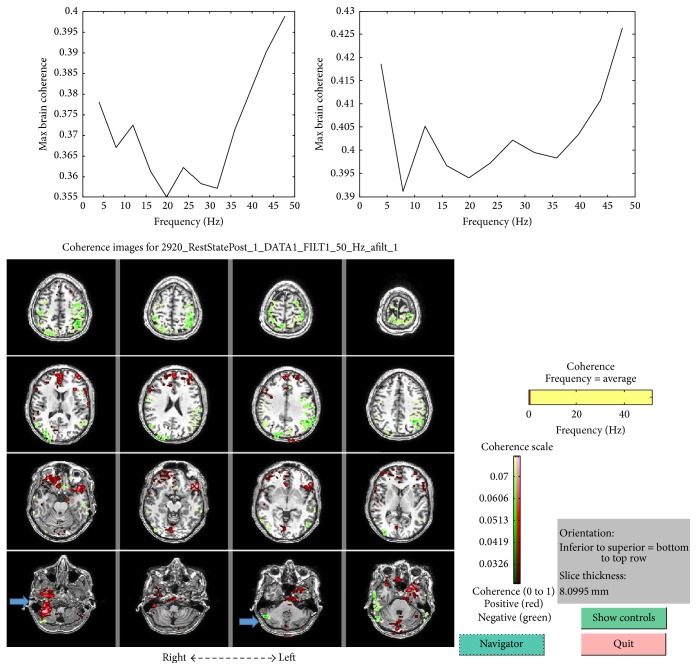
MEG-CSI imaging showing coherence level graphs pretreatment peaks at 13 and 24 Hz (left) and posttreatment peaks at 13 and 28 Hz (right). Pretreatment high coherence is shown in green and posttreatment in red. Arrows indicate cerebellar involvement.

MEG-CSI imaging (3–50 Hz) showed involvement of the right cerebellar hemisphere, premedication state, and peaks in the whole brain coherence in the beta (12.5-30 Hz) and gamma bands (25-100 Hz) (peaks at 13 Hz (COH .37) and 24 Hz (COH .36)). After medication there was involvement of the right and left cerebellar hemisphere and higher whole brain coherence in the beta and gamma bands at 13 Hz (COH .40) and 28 Hz (COH .40), respectively (Figure 1).

**Figure 2:**
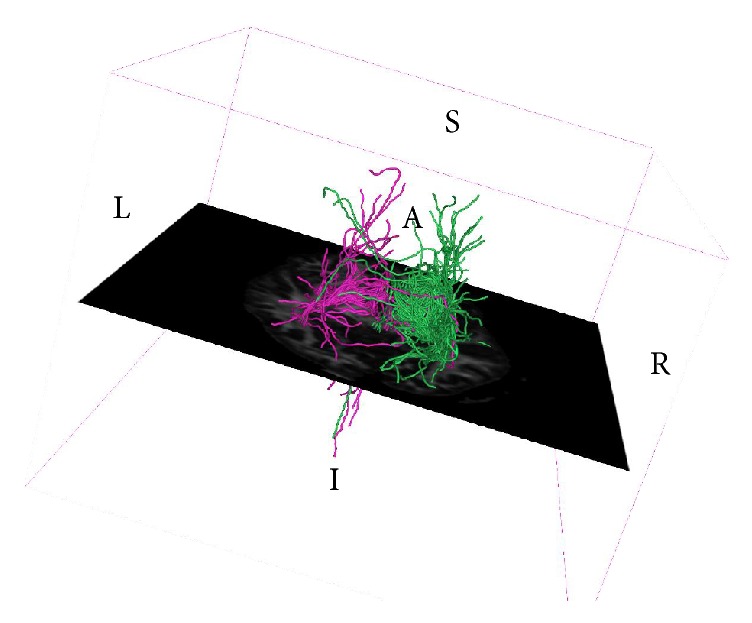
DTI fiber tracking imaging showing fiber tracts indicating stronger connectivity of the right ROI compared to the left to the three cortical areas of interest.

Ipsilateral DTI connectivity was approximately 50% stronger for the right hemisphere compared to the left for each one of the three cortical areas of interest (Figure 2; see also supplementary data for number of fibers reaching the sensory, motor, and premotor cortex, L/R, 62/106, 41/75, 82/121, resp., in Supplementary Material available online at https://doi.org/10.1155/2017/9653520).

## 4. Conclusions

In this case study, microstructural asymmetries as seen on DT correlated well with the clinical phenotype, where there was a left axial tilt, and provide further evidence in support of dystonia as a network disorder [4]. Moreover, the clinically effective contacts (0 and 1 in our patient) did target projections to cortical areas previously identified to be involved in dystonia, like the primary motor and sensory cortex [5–7]. Unilateral pallidal DBS provided considerable clinical improvement, in this patient with asymmetric presentation, as expected and supported by stronger connectivity in the right hemisphere. Furthermore, this is the first study using MEG in hemidystonia. In this study, we have shown (i) asymmetries in functional and structural connectivity, (ii) an increase in whole brain coherence in the beta and gamma bands after medication, and (iii) an involvement of the cerebellum in dystonia as reported before [8, 9]. The increase in gamma band coherence may indicate a partial restoration of intracortical inhibition, typically deficient in patients with dystonia, and local increase in GABA levels, as shown in other studies [10]. Neuronal oscillations during motor movement have been associated with a reduction in alpha (8–12.5 Hz) and beta (12.5–30 Hz) oscillations in EEG activity when subjects made a movement. Brain activity in the 10 Hz rhythms has been recorded with EEG that are linked to cerebellar activation.

Larger case series, in a broader spectrum of clinical phenotypes, utilizing multiparametric imaging could offer further insights into the pathophysiology of dystonia and hence enhance therapeutic approaches.

## Supplementary Material

MEG imaging and coherence graph depicting cerebral activity before and after treatment.
