# Supplementary material for: Multimodal Imaging in a Patient with Hemidystonia Responsive to GPi Deep Brain Stimulation
Source: Case Rep Neurol Med. 2017 Jul 4;2017:9653520. doi: 10.1155/2017/9653520 (PMC5514339; doi:10.1155/2017/9653520)
Supplement: Supplementary file 1 — MEG imaging and coherence graph depicting cerebral activity before and after treatment. [file 9653520.f1.docx]

|  | **Left Hemisphere** | | | | **Right Hemisphere** | | | |
| --- | --- | --- | --- | --- | --- | --- | --- | --- |
| **Cortical Area** | **Sensory** | **Motor** | **Pre-motor** | **Entire** | **Sensory** | **Motor** | **Pre-motor** | **Entire** |
| **Left ROI** | **62** | **41** | **82** | **2991** | **1** | **2** | **0** | **119** |
| **Right ROI** | **18** | **7** | **6** | **197** | **106** | **75** | **121** | **2912** |

Supplementary data demonstrating the number of fibers connecting each ROI to the cortical regions

|  | **Left Hemisphere** | | | | **Right Hemisphere** | | | |
| --- | --- | --- | --- | --- | --- | --- | --- | --- |
| **Cortical Area** | **Sensory** | **Motor** | **Pre-motor** | **Entire** | **Sensory** | **Motor** | **Pre-motor** | **Entire** |
| **Left ROI** | **1.99%** | **1.32%** | **2.64%** | **96.17%** | **0.03%** | **0.06%** | **0%** | **3.83%** |
| **Right ROI** | **0.58%** | **0.23%** | **0.19%** | **6.34%** | **3.41%** | **2.41%** | **3.89%** | **93.64%** |

Supplementary data demonstrating DTI connectivity calculated by dividing the number of fibers of each ROI-cortical connection to the total number of fibers reaching the cortex and then multiplying by 100 to show as percentage
